# Supplementary material for: Ramucirumab and GSK1838705A Enhance the Inhibitory Effects of Low Concentration Sorafenib and Regorafenib Combination on HCC Cell Growth and Motility
Source: Cancers (Basel). 2019 Jun 7;11(6):787. doi: 10.3390/cancers11060787 (PMC6627995; doi:10.3390/cancers11060787)
Supplement: Supplementary file 1 [file cancers-11-00787-s001.pdf]

Supplementary Materials: Ramucirumab and GSK1838705A Enhance the Inhibitory Effects of Low Concentration Sorafenib and Regorafenib Combination on HCC Cell Growth and Motility

Rosalba D'Alessandro, Maria Grazia Refolo, Palma Aurelia Iacovazzi, Pasqua Letizia Pesole, Caterina Messa and Brian Irving Carr

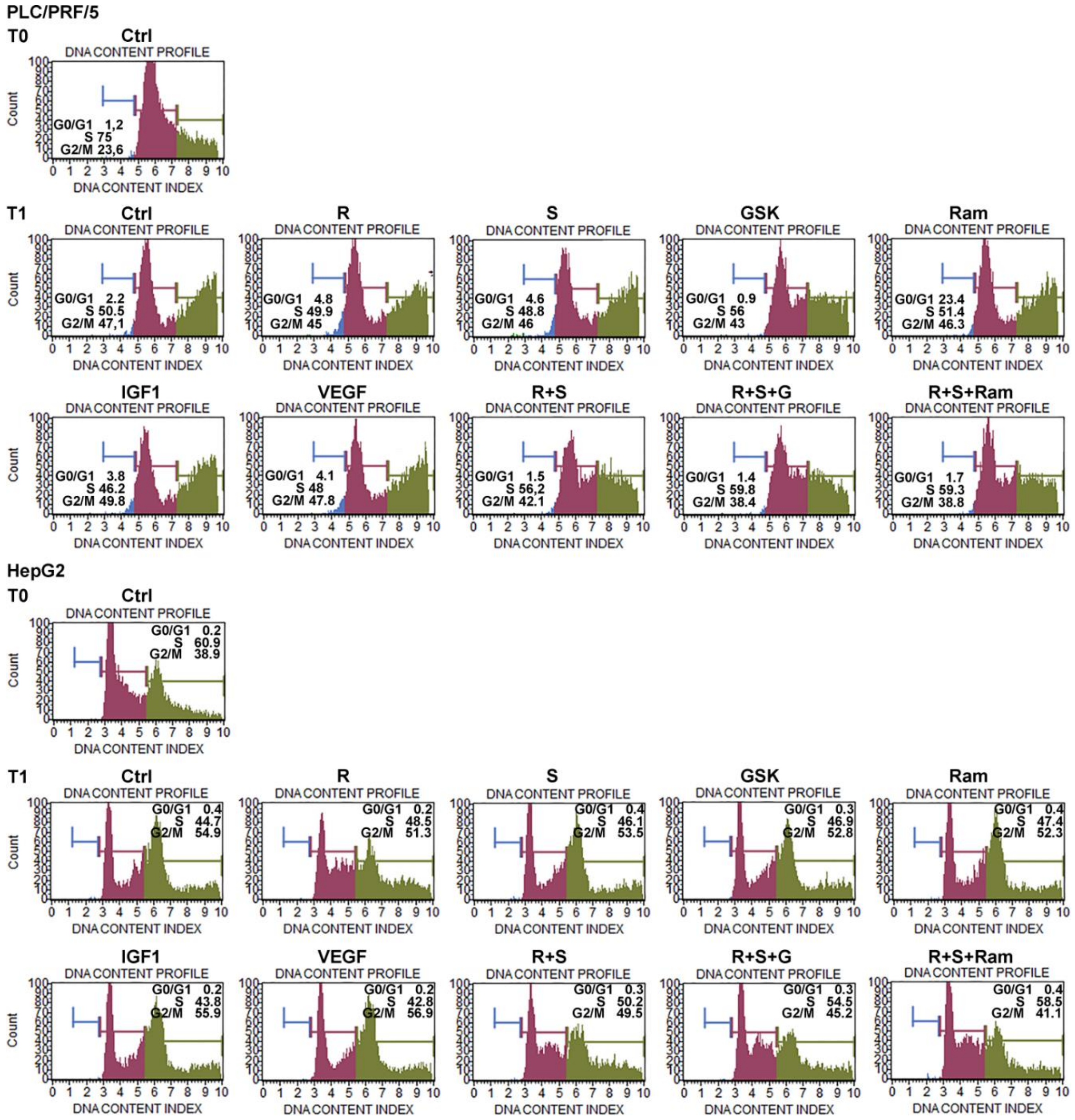

Figure S1. Cell distribution in the cell cycle. Cell cycle analysis performed in cells cultured with 2.5 μM (PLC/PRF/5) or 1 μM (HepG2) Sorafenib (S), 1 μM (PLC/PRF/5) or 0.1 μM (HepG2) Regorafenib (R), 3 μM GSK1838705A (GSK) and 400 μM Ramucirumab (Ram) alone or in combination. IGF1 75 ng/mL and VEGF 20 ng/mL recombinant molecules were used as single treatment. Cells were synchronized with 0.2 M thymidine in the S phase of the cell cycle (T0), after 3 h from block release (T1), the cells were processed and examined with Muse Cell Analyzer to evaluate the percentage of cell distribution in G0/G1, S and G2/M phases. An example of cell cycle progression after different treatments was shown in the panels.

Table S1. Clonogenic Assay. Clonogenic assay performed in cells cultured with 2.5 μM (PLC/PRF/5) or 1 μM (HepG2) Sorafenib (S), 1 μM (PLC/PRF/5) or 0.1 μM (HepG2) Regorafenib (R), 3 μM GSK1838705A (GSK) and 400 μM Ramucirumab (Ram) alone or in combination. IGF1 75 ng/mL and VEGF 20 ng/mL recombinant molecules were used as single treatment. The average value of number of colonies derived from three independent experiments, the *p* value and the percentage decreases for the interest group are reported.

PLC/PRF/5 Samples (Num of Colonies)	"Mann Whitney Test" *p* Value	Decrease (%)
**Ctrl vs. R**<br>94 vs. 64	***<br>0.0001	31.9
**Ctrl vs. S**<br>94 vs. 68.5	***<br>0.0001	27.1
**Ctrl vs. GSK**<br>94 vs. 73	***<br>0.0001	22.3
**Ctrl vs. Ram**<br>94 vs. 76	***<br>0.0001	19.4
**Ctrl vs. IGF**<br>94 vs. 96	ns	2.1
**Ctrl vs. VEGF**<br>94 vs. 101	***<br>0.0001	+7.4
**R vs. R+S**<br>64 vs. 54	***<br>0.0001	15.6
**R vs. R+S+GSK**<br>64 vs. 42	***<br>0.0001	34.4
**R vs. R+S+Ram**<br>64 vs. 32.5	***<br>0.0001	49.2
**S vs. R+S**<br>68.5 vs. 54	***<br>0.0001	21.2
**S vs. R+S+GSK**<br>68.5 vs. 42	***<br>0.0001	38.7
**S vs. R+S+Ram**<br>68.5 vs. 32.5	***<br>0.0001	52.6
**GSK vs. R+S+GSK**<br>73 vs. 42	***<br>0.0001	42.5
**Ram vs. R+S+Ram**<br>76 vs. 32.5	***<br>0.0001	57.2
**R+S vs. R+S+GSK**<br>54 vs. 42	***<br>0.0001	22.2
**R+S vs. R+S+Ram**<br>54 vs. 32.5	***<br>0.0001	39.8
**HepG2 Samples (Num of Colonies)**	**"Mann Whitney Test" *p* Value**	**Decrease (%)**
**Ctrl vs. R**<br>65 vs. 43	***<br>0.0001	33.8
**Ctrl vs. S**<br>65 vs. 49	***<br>0.0001	24.6
**Ctrl vs. GSK**<br>65 vs. 51	***<br>0.0001	21.5
**Ctrl vs. Ram**<br>65 vs. 45	***<br>0.0001	30.8
**Ctrl vs. IGF**<br>65 vs. 70	***<br>0.0001	+7.7
**Ctrl vs. VEGF**<br>65 vs. 59.5	***<br>0.0001	8.5
**R vs. R+S**<br>43 vs. 32.5	***<br>0.0001	24.2

**R vs. R+S+GSK**	***	
43 vs. 24	0.0001	44.2
**R vs. R+S+Ram**	***	
43 vs. 18.5	0.0001	57
**S vs. R+S**	***	
49 vs. 32.5	0.0001	33.7
**S vs. R+S+GSK**	***	
49 vs. 24	0.0001	51
**S vs. R+S+Ram**	***	
49 vs. 18.5	0.0001	62.2
**GSK vs. R+S+GSK**	***	
51 vs. 24	0.0001	52.9
**Ram vs. R+S+Ram**	***	
45 vs. 18.5	0.0001	58.9
**R+S vs. R+S+GSK**	***	
32.5 vs. 24	0.0001	26.2
**R+S vs. R+S+Ram**	***	
32.5 vs. 18.5	0.0001	43.1

Table S2. KI67 immunofluorescent staining. KI67 immunofluorescent staining performed in cells cultured with 2.5 μM (PLC/PRF/5) or 1 μM (HepG2) Sorafenib (S), 1 μM (PLC/PRF/5) or 0.1 μM (HepG2) Regorafenib (R), 3 μM GSK1838705A (GSK) and 400 μM Ramucirumab (Ram) alone or in combination. IGF1 75 ng/mL and VEGF 20 ng/mL recombinant molecules were used as single treatment. The average value of IF intensity signals derived from three independent experiments, the *p* value and the percentage decreases for the interest group are reported.

**PLC/PRF/5 Samples (IF Intensity Signal)**	**"Mann Whitney Test" *p* Value**	**Decrease (%)**
**Ctrl vs. R**	**	
2.3 vs. 2	0.001	13
**Ctrl vs. S**	**	
2.3 vs. 1.7	0.001	26.1
**Ctrl vs. GSK**	***	
2.3 vs. 1.4	0.0001	39.1
**Ctrl vs. Ram**	***	
2.3 vs. 0.9	0.0001	60.9
**Ctrl vs. IGF**	ns	
2.3 vs. 2.6		+13
**Ctrl vs. VEGF**	ns	
2.3 vs. 2.4		+4.3
**R vs. R+S**	***	
2 vs. 1.3	0.0001	35
**R vs. R+S+GSK**	***	
2 vs. 0.5	0.0001	75
**R vs. R+S+Ram**	***	
2 vs. 0.4	0.0001	80
**S vs. R+S**	**	
1.7 vs. 1.3	0.001	23.5
**S vs. R+S+GSK**	***	
1.7 vs. 0.5	0.0001	70.6
**S vs. R+S+Ram**	***	
1.7 vs. 0.4	0.0001	76.5
**GSK vs. R+S+GSK**	***	
1.4 vs. 0.5	0.0001	64.3
**Ram vs. R+S+Ram**	**	
0.9 vs. 0.4	0.001	55.6
**R+S vs. R+S+GSK**	***	

1.3 vs. 0.5	0.0001	61.5
**R+S vs. R+S+Ram**	***	
1.3 vs. 0.4	0.0001	69.2
**HepG2 Samples (IF Intensity Signal)**	**"Mann Whitney Test" *p* Value**	**Decrease (%)**
**Ctrl vs. R**	***	
7.9 vs. 6.5	0.0001	17.7
**Ctrl vs. S**	ns	
7.9 vs. 7.5		5.1
**Ctrl vs. GSK**	ns	
7.9 vs. 7.5		5.1
**Ctrl vs. Ram**	***	
7.9 vs. 6.6	0.0001	16.5
**Ctrl vs. IGF**	ns	
7.9 vs. 8		+1.3
**Ctrl vs. VEGF**	***	
7.9 vs. 11.5	0.0001	+45.6
**R vs. R+S**	**	
6.5 vs. 5.6	0.001	13.8
**R vs. R+S+GSK**	ns	
6.5 vs. 6.3		3.1
**R vs. R+S+Ram**	***	
6.5 vs. 5	0.0001	23.1
**S vs. R+S**	***	
7.5 vs. 5.6	0.0001	25.3
**S vs. R+S+GSK**	***	
7.5 vs. 6.3	0.0001	16
**S vs. R+S+Ram**	***	
7.5 vs. 5	0.0001	33.3
**GSK vs. R+S+GSK**	***	
7.5 vs. 6.3	0.0001	16
**Ram vs. R+S+Ram**	***	
6.6 vs. 5	0.0001	24.2
**R+S vs. R+S+GSK**	**	
5.6 vs. 6.3	0.001	+12.5
**R+S vs. R+S+Ram**	**	
5.6 vs. 5	0.001	10.7

Table S3. Muse Annexin V Cell Assay. Muse Annexin V Cell Assay performed in cells cultured with 2.5 μM (PLC/PRF/5) or 1 μM (HepG2) Sorafenib (S), 1 μM (PLC/PRF/5) or 0.1 μM (HepG2) Regorafenib (R), 3 μM GSK1838705A (GSK) and 400 μM Ramucirumab (Ram) alone or in combination. The average value of the percentage of apoptotic cells derived from three independent experiments, the *p* value and the percentage increases for the interest group are reported.

**PLC/PRF/5 Samples (% of Apoptotic Cells)**	**"Mann Whitney Test" *p* Value**	**Increase (%)**
**Ctrl vs. R**	***	
17.32 vs. 21.67	0.0001	25
**Ctrl vs. S**	***	
17.32 vs. 19.15	0.0001	10.6
**Ctrl vs. GSK**	**	
17.32 vs. 18.16	0.001	4.8
**Ctrl vs. Ram**	***	
17.32 vs. 25.75	0.0001	48.7
**R vs. R+S**	***	
21.67 vs. 23.63	0.0001	9
**R vs. R+S+GSK**	***	

21.67 vs. 30.15	0.0001	39.1
**R vs. R+S+Ram**	***	
21.67 vs. 39.77	0.0001	83.5
**S vs. R+S**	***	
19.15 vs. 23.63	0.0001	23.4
**S vs. R+S+GSK**	***	
19.15 vs. 30.15	0.0001	57.4
**S vs. R+S+Ram**	***	
19.15 vs. 39.77	0.0001	107.7
**GSK vs. R+S+GSK**	***	
18.16 vs. 30.15	0.0001	66
**Ram vs. R+S+Ram**	***	
25.75 vs. 39.77	0.0001	54.4
**R+S vs. R+S+GSK**	***	
23.63 vs. 30.15	0.0001	27.6
**R+S vs. R+S+Ram**	***	
23.63 vs. 39.77	0.0001	68.3
**HepG2 Samples (% of Apoptotic Cells)**	**"Mann Whitney Test" *p* Value**	**Increase (%)**
**Ctrl vs. R**	***	
15.95 vs. 21.55	0.0001	35.1
**Ctrl vs. S**	***	
15.95 vs. 21.17	0.0001	32.7
**Ctrl vs. GSK**	***	
15.95 vs. 21.52	0.0001	34.9
**Ctrl vs. Ram**	***	
15.95 vs. 28.23	0.0001	77
**R vs. R+S**	***	
21.55 vs. 27.88	0.0001	29.4
**R vs. R+S+GSK**	***	
21.55 vs. 26.17	0.0001	21.4
**R vs. R+S+Ram**	***	
21.55 vs. 38.53	0.0001	78.8
**S vs. R+S**	***	
21.17 vs. 27.88	0.0001	31.7
**S vs. R+S+GSK**	***	
21.17 vs. 26.17	0.0001	23.6
**S vs. R+S+Ram**	***	
21.17 vs. 38.53	0.0001	82
**GSK vs. R+S+GSK**	***	
21.52 vs. 26.17	0.0001	21.6
**Ram vs. R+S+Ram**	***	
28.23 vs. 38.53	0.0001	36.5
**R+S vs. R+S+GSK**	ns	
27.88 vs. 26.17		-0.9
**R+S vs. R+S+Ram**	***	
27.88 vs. 38.53	0.0001	38.2

Table S4. Cell Migration Assay. Cell Migration Assay performed in cells cultured with 2.5 µM (PLC/PRF/5) or 1 µM (HepG2) Sorafenib (S), 1 µM (PLC/PRF/5) or 0.1 µM (HepG2) Regorafenib (R), 3 µM GSK1838705A (GSK) and 400 µM Ramucirumab (Ram) alone or in combination. The average value of the percentage of migration derived from three independent experiments, the *p* value and the percentage decreases for the interest group are reported.

PLC/PRF/5 Samples (% of Migration)	"Mann Whitney Test" *p* Value	Decrease (%)
**Ctrl vs. R**<br>57.09 vs. 29.17	***<br>0.0001	48.9
**Ctrl vs. S**<br>57.09 vs. 19.65	***<br>0.0001	65.6
**Ctrl vs. GSK**<br>57.09 vs. 45.59	***<br>0.0001	20.1
**Ctrl vs. Ram**<br>57.09 vs. 27.19	***<br>0.0001	52.4
**R vs. R+S**<br>29.17 vs. 13.59	***<br>0.0001	53.4
**R vs. R+S+GSK**<br>29.17 vs. 8.06	***<br>0.0001	72.4
**R vs. R+S+Ram**<br>29.17 vs. 3.59	***<br>0.0001	87.7
**S vs. R+S**<br>19.65 vs. 13.59	***<br>0.0001	30.8
**S vs. R+S+GSK**<br>19.65 vs. 8.06	***<br>0.0001	59
**S vs. R+S+Ram**<br>19.65 vs. 3.59	***<br>0.0001	81.7
**GSK vs. R+S+GSK**<br>45.59 vs. 8.06	***<br>0.0001	82.3
**Ram vs. R+S+Ram**<br>27.19 vs. 3.59	***<br>0.0001	99.9
**R+S vs. R+S+GSK**<br>13.59 vs. 8.06	***<br>0.0001	40.7
**R+S vs. R+S+Ram**<br>13.59 vs. 3.59	***<br>0.0001	73.6
**HepG2 Samples (% of Migration)**	**"Mann Whitney Test" *p* Value**	**Decrease (%)**
**Ctrl vs. R**<br>17.40 vs. 6.59	***<br>0.0001	62.1
**Ctrl vs. S**<br>17.40 vs. 5.93	***<br>0.0001	65.9
**Ctrl vs. GSK**<br>17.40 vs. 10.55	***<br>0.0001	39.4
**Ctrl vs. Ram**<br>17.40 vs. 0.94	***<br>0.0001	94.6
**R vs. R+S**<br>6.59 vs. 5.91	ns	10.3
**R vs. R+S+GSK**<br>6.59 vs. 3.11	***<br>0.0001	52.8
**R vs. R+S+Ram**<br>6.59 vs. 0.56	***<br>0.0001	91.5
**S vs. R+S**<br>5.93 vs. 5.91	ns	0.3
**S vs. R+S+GSK**<br>5.93 vs. 3.11	***<br>0.0001	47.6
**S vs. R+S+Ram**<br>5.93 vs. 0.56	***<br>0.0001	90.6

**GSK vs. R+S+GSK**	***	
10.55 vs. 3.11	0.0001	70.5
**Ram vs. R+S+Ram**	***	
0.94 vs. 0.56	0.0001	40.4
**R+S vs. R+S+GSK**	***	
5.91 vs. 3.11	0.0001	47.4
**R+S vs. R+S+Ram**	***	
5.91 vs. 0.56	0.0001	90.5

Table S5. DyLight 554 Phalloidin immunofluorescent staining. DyLight 554 Phalloidin immunofluorescent staining performed in cells cultured with 2.5 μM (PLC/PRF/5) or 1 μM (HepG2) Sorafenib (S), 1 μM (PLC/PRF/5) or 0.1 μM (HepG2) Regorafenib (R), 3 μM GSK1838705A (GSK) and 400 μM Ramucirumab (Ram) alone or in combination. IGF1 75 ng/mL and VEGF 20 ng/mL recombinant molecules were used as single treatment. The average value of IF intensity signals derived from three independent experiments, the *p* value and the percentage decreases for the interest group are reported.

**PLC/PRF/5 Samples (IF Intensity Signal)**	**"Mann Whitney Test" *p* Value**	**Decrease (%)**
**Ctrl vs. R**	***	
13.8 vs. 6.1	0.0001	55.8
**Ctrl vs. S**	***	
13.8 vs. 7.2	0.0001	47.8
**Ctrl vs. GSK**	***	
13.8 vs. 5.8	0.0001	58
**Ctrl vs. Ram**	***	
13.8 vs. 8.5	0.0001	38.4
**Ctrl vs. IGF**	ns	
13.8 vs. 13.9		+0.8
**Ctrl vs. VEGF**	**	
13.8 vs. 11	0.001	20.8
**R vs. R+S**	***	
6.1 vs. 0.9	0.0001	85.2
**R vs. R+S+GSK**	***	
6.1 vs. 0.2	0.0001	96.7
**R vs. R+S+Ram**	***	
6.01 vs. 0.9	0.0001	85
**S vs. R+S**	***	
7.2 vs. 0.9	0.0001	87.5
**S vs. R+S+GSK**	***	
7.2 vs. 0.2	0.0001	97.2
**S vs. R+S+Ram**	***	
7.2 vs. 0.9	0.0001	87.5
**GSK vs. R+S+GSK**	***	
5.8 vs. 0.2	0.0001	96.6
**Ram vs. R+S+Ram**	***	
8.5 vs. 0.9	0.0001	89.4
**R+S vs. R+S+GSK**	***	
0.9 vs. 0.2	0.0001	77.8
**R+S vs. R+S+Ram**	ns	
0.9 vs. 0.9		0
**HepG2 Samples (IF Intensity Signal)**	**"Mann Whitney Test" *p* Value**	**Decrease (%)**
**Ctrl vs. R**	***	
20 vs. 12.2	0.0001	39
**Ctrl vs. S**	***	
20 vs. 14	0.0001	30

**Ctrl vs. GSK**<br>20 vs. 14.2	***<br>0.0001	29
**Ctrl vs. Ram**<br>20 vs. 15	***<br>0.0001	25
**Ctrl vs. IGF**<br>20 vs. 19.2	ns	4
**Ctrl vs. VEGF**<br>20 vs. 19.2	ns	4
**R vs. R+S**<br>12.2 vs. 10.1	ns	17.2
**R vs. R+S+GSK**<br>12.2 vs. 7.1	***<br>0.0001	41.8
**R vs. R+S+Ram**<br>12.2 vs. 6.2	***<br>0.0001	49.2
**S vs. R+S**<br>14 vs. 10.1	**<br>0.001	27.9
**S vs. R+S+GSK**<br>14 vs. 7.1	***<br>0.0001	49.3
**S vs. R+S+Ram**<br>14 vs. 6.2	***<br>0.0001	55.7
**GSK vs. R+S+GSK**<br>14.2 vs. 7.1	***<br>0.0001	50
**Ram vs. R+S+Ram**<br>15 vs. 6.2	***<br>0.0001	58.7
**R+S vs. R+S+GSK**<br>10.1 vs. 7.1	**<br>0.001	29.7
**R+S vs. R+S+Ram**<br>10.1 vs. 6.2	**<br>0.001	38.6

Table S6. (A) AFP levels. Chemiluminescent immunometric assay performed in cells cultured with 2.5 μM (PLC/PRF/5) or 1 μM (HepG2) Sorafenib (S), 1 μM (PLC/PRF/5) or 0.1 μM (HepG2) Regorafenib (R), 3 μM GSK1838705A (GSK) and 400 μM Ramucirumab (Ram) alone or in combination. The average value of AFP/cell derived from three independent experiments, the *p* value and the percentage decreases for the interest group are reported.

**PLC/PRF/5 Samples (AFP/Cell)**	**"Mann Whitney Test" *p* Value**	**Decrease (%)**
**Ctrl vs. R**<br>1 vs. 0.82	***<br>0.0001	18
**Ctrl vs. S**<br>1 vs. 0.72	***<br>0.0001	28
**Ctrl vs. GSK**<br>1 vs. 0.79	***<br>0.0001	21
**Ctrl vs. Ram**<br>1 vs. 0.73	***<br>0.0001	27
**R vs. R+S**<br>0.82 vs. 0.53	***<br>0.0001	35.4
**R vs. R+S+GSK**<br>0.82 vs. 0.29	***<br>0.0001	64.6
**R vs. R+S+Ram**<br>0.82 vs. 0.49	***<br>0.0001	40.2
**S vs. R+S**<br>0.72 vs. 0.53	***<br>0.0001	26.4
**S vs. R+S+GSK**<br>0.72 vs. 0.29	***<br>0.0001	59.7
**S vs. R+S+Ram**<br>0.72 vs. 0.49	***<br>0.0001	31.9

**GSK vs. R+S+GSK**	***	
0.79 vs. 0.29	0.0001	63.3
**Ram vs. R+S+Ram**	***	
0.73 vs. 0.49	0.0001	32.9
**R+S vs. R+S+GSK**	***	
0.53 vs. 0.29	0.0001	45.3
**R+S vs. R+S+Ram**	***	
0.53 vs. 0.49	0.0001	7.5
**HepG2 Samples (AFP/Cell)**	**"Mann Whitney Test" *p* Value**	**Decrease (%)**
**Ctrl vs. R**	***	
1 vs. 0.63	0.0001	37
**Ctrl vs. S**	**	
1 vs. 0.79	0.001	21
**Ctrl vs. GSK**	***	
1 vs. 0.81	0.0001	19
**Ctrl vs. Ram**	***	
1 vs. 0.79	0.0001	21
**R vs. R+S**	ns	
0.63 vs. 0.66		+4.8
**R vs. R+S+GSK**	***	
0.63 vs. 0.56	0.0001	11.1
**R vs. R+S+Ram**	***	
0.63 vs. 0.41	0.0001	34.9
**S vs. R+S**	***	
0.79 vs. 0.66	0.0001	16.5
**S vs. R+S+GSK**	***	
0.79 vs. 0.56	0.0001	29.1
**S vs. R+S+Ram**	***	
0.79 vs. 0.41	0.0001	48.1
**GSK vs. R+S+GSK**	***	
0.81 vs. 0.56	0.0001	30.9
**Ram vs. R+S+Ram**	***	
0.79 vs. 0.41	0.0001	48.1
**R+S vs. R+S+GSK**	***	
0.66 vs. 0.56	0.0001	15.2
**R+S vs. R+S+Ram**	***	
0.66 vs. 0.41	0.0001	37.9

Table S6. (B). DCP levels. Chemiluminescent immunometric assay performed in cells cultured with 2.5 μM (PLC/PRF/5) or 1 μM (HepG2) Sorafenib (S), 1 μM (PLC/PRF/5) or 0.1 μM (HepG2) Regorafenib (R), 3 μM GSK1838705A (GSK) and 400 μM Ramucirumab (Ram) alone or in combination. The average value of DCP/cell derived from three independent experiments, the *p* value and the percentage decreases for the interest group are reported.

PLC/PRF/5 Samples (DCP/Cell)	"Mann Whitney Test" *p* Value	Decrease (%)
**Ctrl vs. R**	***	
1 vs. 0.69	0.0001	31
**Ctrl vs. S**	***	
1 vs. 0.61	0.0001	39
**Ctrl vs. GSK**	***	
1 vs. 0.64	0.0001	36
**Ctrl vs. Ram**	ns	
1 vs. 1.18		+18
**R vs. R+S**	***	
0.69 vs. 0.19	0.0001	72.5

**R vs. R+S+GSK** 0.69 vs. 0.12	*** 0.0001	82.6
**R vs. R+S+Ram** 0.69 vs. 0.23	*** 0.0001	66.7
**S vs. R+S** 0.61 vs. 0.19	*** 0.0001	68.9
**S vs. R+S+GSK** 0.61 vs. 0.12	*** 0.0001	80.3
**S vs. R+S+Ram** 0.61 vs. 0.23	*** 0.0001	62.3
**GSK vs. R+S+GSK** 0.64 vs. 0.12	*** 0.0001	81.3
**Ram vs. R+S+Ram** 1.18 vs. 0.23	*** 0.0001	80.5
**R+S vs. R+S+GSK** 0.19 vs. 0.12	*** 0.0001	36.8
**R+S vs. R+S+Ram** 0.19 vs. 0.23	ns	+21.1
**HepG2 Samples (DCP/Cell)**	**"Mann Whitney Test" *p* Value**	**Decrease (%)**
**Ctrl vs. R** 1 vs. 0.82	** 0.001	18
**Ctrl vs. S** 1 vs. 0.99	ns	1
**Ctrl vs. GSK** 1 vs. 1.01	ns	+1
**Ctrl vs. Ram** 1 vs. 0.72	*** 0.0001	28
**R vs. R+S** 0.82 vs. 0.79	ns	3.7
**R vs. R+S+GSK** 0.82 vs. 0.79	ns	3.7
**R vs. R+S+Ram** 0.82 vs. 0.59	*** 0.0001	28
**S vs. R+S** 0.99 vs. 0.79	*** 0.0001	20
**S vs. R+S+GSK** 0.99 vs. 0.79	*** 0.0001	20
**S vs. R+S+Ram** 0.99 vs. 0.59	*** 0.0001	40.4
**GSK vs. R+S+GSK** 1.01 vs. 0.79	*** 0.0001	21.8
**Ram vs. R+S+Ram** 0.72 vs. 0.59	*** 0.0001	18.1
**R+S vs. R+S+GSK** 0.79 vs. 0.79	ns	0
**R+S vs. R+S+Ram** 0.79 vs. 0.59	*** 0.0001	25.3

Table S7. (A) Muse MAPK Activation Dual Detection Assay. Muse MAPK Activation Dual Detection Assay performed in cells cultured with 2.5 μM (PLC/PRF/5) or 1 μM (HepG2) Sorafenib (S), 1 μM (PLC/PRF/5) or 0.1 μM (HepG2) Regorafenib (R), 3 μM GSK1838705A (GSK) and 400 μM Ramucirumab (Ram) alone or in combination. The average value of percentage of P-Erk activated cells derived from three independent experiments, the *p* value and the percentage decreases for the interest group are reported.

**PLC/PRF/5 Samples (% P-Erk Activated Cells)**	**"Mann Whitney Test" *p* Value**	**Decrease (%)**
**Ctrl vs. R**	***	
86.67 vs. 77.67	0.0001	10.4
**Ctrl vs. S**	***	
86.67 vs. 79.67	0.0001	8
**Ctrl vs. GSK**	***	
86.67 vs. 68	0.0001	21.5
**Ctrl vs. Ram**	***	
86.67 vs. 75	0.0001	13.5
**R vs. R+S**	***	
77.67 vs. 68.33	0.0001	12
**R vs. R+S+GSK**	***	
77.67 vs. 63	0.0001	18.9
**R vs. R+S+Ram**	***	
77.67 vs. 54.33	0.0001	30.1
**S vs. R+S**	***	
79.67 vs. 68.33	0.0001	14.2
**S vs. R+S+GSK**	***	
79.67 vs. 68	0.0001	14.6
**S vs. R+S+Ram**	***	
79.67 vs. 54.33	0.0001	31.8
**GSK vs. R+S+GSK**	**	
68 vs. 63	0.001	4.4
**Ram vs. R+S+Ram**	***	
75 vs. 54.33	0.0001	27.6
**R+S vs. R+S+GSK**	**	
68.33 vs. 63	0.001	7.8
**R+S vs. R+S+Ram**	***	
68.33 vs. 54.33	0.0001	20.5
**HepG2 Samples (% P-Erk Activated Cells)**	**"Mann Whitney Test" *p* Value**	**Decrease (%)**
**Ctrl vs. R**	***	
97.03 vs. 85.63	0.0001	11.7
**Ctrl vs. S**	***	
97.03 vs. 89.27	0.0001	8
**Ctrl vs. GSK**	***	
97.03 vs. 91.90	0.0001	5.3
**Ctrl vs. Ram**	***	
97.03 vs. 90.53	0.0001	6.7
**R vs. R+S**	***	
85.63 vs. 77.97	0.0001	8.9
**R vs. R+S+GSK**	***	
85.63 vs. 65.07	0.0001	24
**R vs. R+S+Ram**	***	
85.63 vs. 63.83	0.0001	25.5
**S vs. R+S**	***	
89.27 vs. 77.97	0.0001	12.7
**S vs. R+S+GSK**	***	
89.27 vs. 65.07	0.0001	27.1

**S vs. R+S+Ram**	***	
89.27 vs. 63.83	0.0001	28.5
**GSK vs. R+S+GSK**	***	
91.90 vs. 65.07	0.0001	29.2
**Ram vs. R+S+Ram**	***	
90.53 vs. 63.83	0.0001	29.5
**R+S vs. R+S+GSK**	***	
77.97 vs. 65.07	0.0001	16.5
**R+S vs. R+S+Ram**	***	
77.97 vs. 63.83	0.0001	18.1

Table S7. (**B**) Muse PI3K Activation Dual Detection Assay. Muse PI3K Activation Dual Detection Assay performed in cells cultured with 2.5 µM (PLC/PRF/5) or 1 µM (HepG2) Sorafenib (S), 1 µM (PLC/PRF/5) or 0.1 µM (HepG2) Regorafenib (R), 3 µM GSK1838705A (GSK) and 400 µM Ramucirumab (Ram) alone or in combination. The average value of percentage of P-Akt activated cells derived from three independent experiments, the *p* value and the percentage decreases for the interest group are reported.

PLC/PRF/5 Samples (% P-Akt Activated Cells)	"Mann Whitney Test" *p* Value	Decrease (%)
**Ctrl vs. R**	***	
86.33 vs. 81.97	0.0001	5.1
**Ctrl vs. S**	*	
86.33 vs. 84	0.05	2.7
**Ctrl vs. GSK**	***	
86.33 vs. 68.13	0.0001	21.1
**Ctrl vs. Ram**	***	
86.33 vs. 68.47	0.0001	20.7
**R vs. R+S**	***	
81.97 vs. 69.83	0.0001	14.7
**R vs. R+S+GSK**	***	
81.97 vs. 56.70	0.0001	30.8
**R vs. R+S+Ram**	***	
81.97 vs. 52.60	0.0001	35.8
**S vs. R+S**	***	
84 vs. 69.83	0.0001	16.9
**S vs. R+S+GSK**	***	
84 vs. 56.70	0.0001	32.5
**S vs. R+S+Ram**	***	
84 vs. 52.60	0.0001	37.4
**GSK vs. R+S+GSK**	***	
68.13 vs. 56.70	0.0001	16.8
**Ram vs. R+S+Ram**	***	
68.47 vs. 52.60	0.0001	23.2
**R+S vs. R+S+GSK**	***	
69.83 vs. 56.70	0.0001	18.8
**R+S vs. R+S+Ram**	***	
69.83 vs. 52.60	0.0001	24.7
**HepG2 Samples (% P-Akt Activated Cells)**	**"Mann Whitney Test" *p* Value**	**Decrease (%)**
**Ctrl vs. R**	***	
92.17 vs. 84.07	0.0001	8.8
**Ctrl vs. S**	***	
92.17 vs. 84.97	0.0001	7.8
**Ctrl vs. GSK**	***	
92.17 vs. 64.70	0.0001	29.8
**Ctrl vs. Ram**	***	

92.17 vs. 55.53	0.0001	39.8
**R vs. R+S**	***	
84.07 vs. 76.07	0.0001	9.5
**R vs. R+S+GSK**	***	
84.07 vs. 44.93	0.0001	46.6
**R vs. R+S+Ram**	***	
84.07 vs. 37.10	0.0001	55.9
**S vs. R+S**	***	
84.97 vs. 76.07	0.0001	10.5
**S vs. R+S+GSK**	***	
84.97 vs. 44.93	0.0001	47.1
**S vs. R+S+Ram**	***	
84.97 vs. 37.10	0.0001	56.3
**GSK vs. R+S+GSK**	***	
64.70 vs. 44.93	0.0001	30.6
**Ram vs. R+S+Ram**	***	
55.53 vs. 37.10	0.0001	33.2
**R+S vs. R+S+GSK**	***	
76.07 vs. 44.93	0.0001	40.9
**R+S vs. R+S+Ram**	***	
76.07 vs. 37.10	0.0001	51.2

© 2019 by the authors. Licensee MDPI, Basel, Switzerland. This article is an open access article distributed under the terms and conditions of the Creative Commons Attribution (CC BY) license (http://creativecommons.org/licenses/by/4.0/).
